# Quantifying the Contribution of Hosts with Different Parasite Concentrations to the Transmission of Visceral Leishmaniasis in Ethiopia

**DOI:** 10.1371/journal.pntd.0003288

**Published:** 2014-10-30

**Authors:** Ezer Miller, Alon Warburg, Ilya Novikov, Asrat Hailu, Petr Volf, Veronika Seblova, Amit Huppert

**Affiliations:** 1 The Kuvin Center for the Study of Infectious & Tropical Diseases, Department of Microbiology & Molecular Genetics, The Institute of Medical Research, Israel-Canada, Faculty of Medicine, The Hebrew University of Jerusalem, Jerusalem, Israel; 2 The Biostatistics Unit, The Gertner Institute for Epidemiology and Health Policy Research, Sheba Medical Center, Tel Hashomer, Ramat Gan, Israel; 3 Department of Microbiology, Immunology & Parasitology, Faculty of Medicine, Addis Ababa University, Addis Ababa, Ethiopia; 4 Department of Parasitology, Faculty of Sciences, Charles University, Prague, Czech Republic; Fundaçao Oswaldo Cruz, Brazil

## Abstract

**Background:**

An important factor influencing the transmission dynamics of vector-borne diseases is the contribution of hosts with different parasitemia (no. of parasites per ml of blood) to the infected vector population. Today, estimation of this contribution is often impractical since it relies exclusively on limited-scale xenodiagnostic or artificial feeding experiments (i.e., measuring the proportion of vectors that become infected after feeding on infected blood/host).

**Methodology:**

We developed a novel mechanistic model that facilitates the quantification of the contribution of hosts with different parasitemias to the infection of the vectors from data on the distribution of these parasitemias within the host population. We applied the model to an ample data set of *Leishmania donovani* carriers, the causative agent of visceral leishmaniasis in Ethiopia.

**Results:**

Calculations facilitated by the model quantified the host parasitemias that are mostly responsible for the infection of vector, the sand fly *Phlebotomus orientalis*. Our findings indicate that a 3.2% of the most infected people were responsible for the infection of between 53% and 79% (mean – 62%) of the infected sand fly vector population.

**Significance:**

Our modeling framework can easily be extended to facilitate the calculation of the contribution of other host groups (such as different host species, hosts with different ages) to the infected vector population. Identifying the hosts that contribute most towards infection of the vectors is crucial for understanding the transmission dynamics, and planning targeted intervention policy of visceral leishmaniasis as well as other vector borne infectious diseases (e.g., West Nile Fever).

## Author Summary

An important factor influencing the transmission dynamics of vector-borne diseases is the contribution of hosts with different parasitemia (no. of parasites per ml of blood) to the infected vector population. In this study we developed a novel mechanistic model that facilitates the quantification of this contribution and applied it to an ample data set of people infected with visceral leishmaniasis in Ethiopia. Among vector borne diseases, visceral leishmaniasis is the second most important killer after malaria. It is caused by infection with *Leishmania* parasites with most cases (∼90%) occurring in the Indian sub-continent, East Africa, and South America. The disease is transmitted between people and other mammalian hosts by blood-sucking sand flies. Our findings indicate that a 3.2% of the most infected people were responsible for the infection of about 65% of the infected sand fly vector population. Identifying the hosts that contribute most towards infection of the vectors is crucial for understanding the transmission dynamics, and planning targeted intervention policy of visceral leishmaniasis, as well as other vector borne infectious diseases (e.g., Dengue, West Nile Fever).

## Introduction

An important factor influencing the transmission dynamics of Vector-Borne Diseases (VBDs) is the contribution of hosts with different parasitemia (no. of parasites per ml of blood) to the infected vector population (the Host Infectiousness Profile, HIP) [1], [2]. Identifying the hosts that contribute most to the infection of the vectors is crucial for understanding the transmission dynamics of VBDs, as well as for planning intervention strategies targeting the relevant infected host groups [1], [2]. Calculating this contribution demands quantitative information on the distribution of different parasitemias within the host population and their infectiousness to the vectors. Quantifying the distribution of hosts with different parasitemias within the population is usually achieved through mass screening using molecular, parasitological, or immunological approaches [3],[4],[5]. Today, quantifying the infectiousness of hosts with different parasitemias relies on xenodiagnosis or artificial infection experiments which directly determine the infectiousness of the host blood by measuring the proportion of vectors that become infected after feeding on it. However, such experiments can be prohibitively expensive, and require experienced staff and adequate insectary facilities. Moreover, xenodiagnosis on human volunteers is frequently disallowed by institutional ethical review boards or Helsinki committees. Xenodiagnosis or artificial infection experiments are therefore limited to a small number of both, hosts and vectors [2], [4], [6]. Thus, efficient estimation of host infectiousness is hindered by small sample sizes. Clearly, a quantitative mathematical model designed to interpolate or extrapolate the infectiousness of hosts with different parasitemias without necessitating direct measurement, would constitute a crucial addition to our disease-fighting arsenal. In this study we develop such a mechanistic model, the first of its kind, that enables the calculation of HIP (Host Infectiousness Profile) from given host parasitemia distribution for a wide range of VBDs, and apply it to a data set of human volunteers, potential hosts of Visceral Leishmaniasis (VL) in northern Ethiopia [3].

Among VBDs, VL is the second most important killer (500,000 cases and 70,000 deaths annually) after malaria [7], [8]. VL is caused by infection with *Leishmania* ssp. with most cases (∼90%) occurring in the Indian sub-continent, East Africa, and South America [7], [9]. The sand fly (Diptera: Psychodidae) females become infected when they bite infectious humans (anthroponotic transmission) or other mammalian hosts (zoonotic transmission) [7], [8]. The *Leishmania* parasites proliferate in the lumen of its gut and are transmitted to a naïve host when the sand fly female imbibes a subsequent blood-meal [7], [8]. Although most of the people infected with VL remain free of disease, they may be infectious to biting sand flies and, thereby, contribute to the propagation of the disease. Yet, the potential role of these asymptomatic carriers to the transmission remains largely unknown [10], [11], [12]. The main goal of the current study was to estimate the contribution of *L. donovani* carriers with different blood parasitemias to the infection of *Phlebotomus orientalis*, the sand fly vector of VL in northern Ethiopia, by developing a customized mathematical model.

## Methods

To achieve the study goal, we first developed an infectiousness model aiming at quantifying the host infectiousness to biting sand flies as a function of its parasitemia. We then used this model to calculate the HIP from the data on the distribution of parasitemias in the host population obtained through cohort study [3].

### Host Infectiousness Model

To develop the infectiousness model, we assumed that the infectiousness of a host for a biting insect depends exclusively on its blood parasitemia (although parasites may exist in other tissues, such as the spleen or the skin, e.g., VL [13]). Following the above assumption, let *n*, be the blood parasitemia of an infected host. The probability that *j* parasites will be ingested by a vector imbibing volume *v* of blood per bite form an infected host, *r*(*j*), is Poisson distributed (where *nv* is its mean),
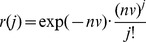
(1)


We assume that the infection process within the vector is density-independent (i.e., the probability, *p*, that a single parasite will infect a vector is a fixed value independent of the presence of other parasites). Thus, each parasite is equally infectious regardless of the number of parasites ingested. Indeed, the number of *Leishmania* parasites at the initial stages of the sand fly infection is usually very low (e.g., between 1–500), while sand flies with mature infection frequently harbor tens of thousands of parasites [14], [15], [16]. Once the infection in the vector gut has been established through this density-independent process, the progression (that may be density-dependent due to intraspecific competition) to mature transmissible infection, is thought to be almost definite [14], [15], [16]. Thus, the probability that the *j* parasites in equation (1) will infect the vector, *s*(*j*), is given by:
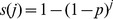
(2)


Since the number of parasites ingested during a single blood meal and the infectiousness of any one of these parasites are independent random variables, we have:
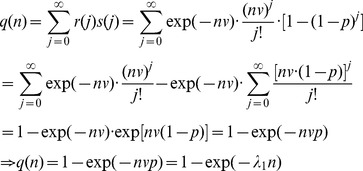
(3)Where *q*(*n*) in equation (3) is the infectious probability (i.e., the probability that the vector will be infected after biting a host with parasitemia *n*), and λ_1_ = *vp*, is the probability that a host with 1 parasite per ml of blood will infect the vector. Empirical studies have demonstrated that a certain proportion of the vectors do not become infected irrespective of the number of parasites ingested during feeding (i.e., irrespective of *n*), due to incompatible gut microbiota, for example [17], [18], [19]. Let 1-λ_2_, be that proportion. By assuming that the infectious probability of the host, *q*(*n*), and the probability that a vector is able to become infected, λ_2_, are independent, we have:
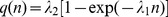
(4)


Equation (4) represents the infectiousness of a host with parasitemia, *n*, for a feeding vector. Note that equation (4) depends on two parameters (λ_1_, λ_2_), which vary according to vector, host, disease type, and environmental parameters (e.g., temperature) [20]. This equation is our key result since it enables the calculation of the HIP from the distribution of host parasitemias.

### Cohort Study

As part of a cohort study aiming at improving our understanding of the transmission dynamics of VL, blood samples were collected from *N* = 4,695 villagers in the Sheraro district of northern Ethiopia. A highly sensitive quantitative real-time PCR was performed to detect *Leishmania* kinetoplast DNA (kDNA) in dried blood samples [3]. These results were used to obtain the proportion of various parasitemia ranges within the population. The errors in these proportions were calculated according to the variance of a multinomial distribution.

### The Contribution of Infected Hosts Belonging to Different Parasitemia Categories to the Infected Vector Population

Once the data on the distribution of parasitemias in the host population is obtained, it is possible to calculate, with the aid of equation (4), the contribution of hosts with different parasitemia levels to the infected vector population, i.e., the HIP. First, an estimation of the model parameters (λ_1_ and λ_2_, equation (4)) should be performed by fitting the model (equation (4)) to data obtained from artificial infection experiments. Provided that all hosts have an equal probability of being bitten, the calculation of the contribution of hosts with different parasitemia ranges to the infected vector population is straightforward; Let *z*_1_ and *z*_2_, *z*_1_<z_2_, denote two parasite concentrations, and *m* denote the number of hosts with parasitemias between *z*_1_ and *z*_2_. If *I*_1→2_ is the proportion of vectors (from all infected vectors) that were infected by biting hosts with parasitemias between *z*_1_ and *z*_2_, then:
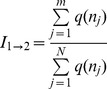
(5)


In equation (5), the numerator sums the *q*(*n*_j_) (the probability that the vector will be infected by biting a host with parasitemia *n_j_*, equation (4)) over all *m* hosts for which parasite concentrations are between *z*_1_ and *z*_2_. The denumerator sums the *q*(*n_j_*) for the entire cohort,(i.e., *N* = population size = 4,695 in our case). Previous studies indicate that the vector biting rate is not uniform among the host population but depends on the host's specific volatiles that may cause sick hosts, for example, to be more attractive for the vectors [21], [22], [23], [24]. We assumed uniform biting rates in our model since most people infected with *L. donovani* remain asymptomatic (i.e., disease free) and are, therefore, presumed not to emit volatiles associated with sickness [8]. Thus, as long as the vectors' preference is not affected by host parasitemia per se, equation (5) remains valid. A more general version of equation (5) which takes into account putative vector preferences is discussed below in equation (6). Note that although equation (4) was developed to calculate the probability, *q*(*n*), of a vector being infected after a single bite, equation (5) holds true irrespective of the number of bites the vector delivers through its life. The reason is that the HIP is determined exclusively by naïve vectors, that acquire their infection only once (infected vectors remain infected till they die).

## Results

### Host Infectiousness Model

**Figure 1:**
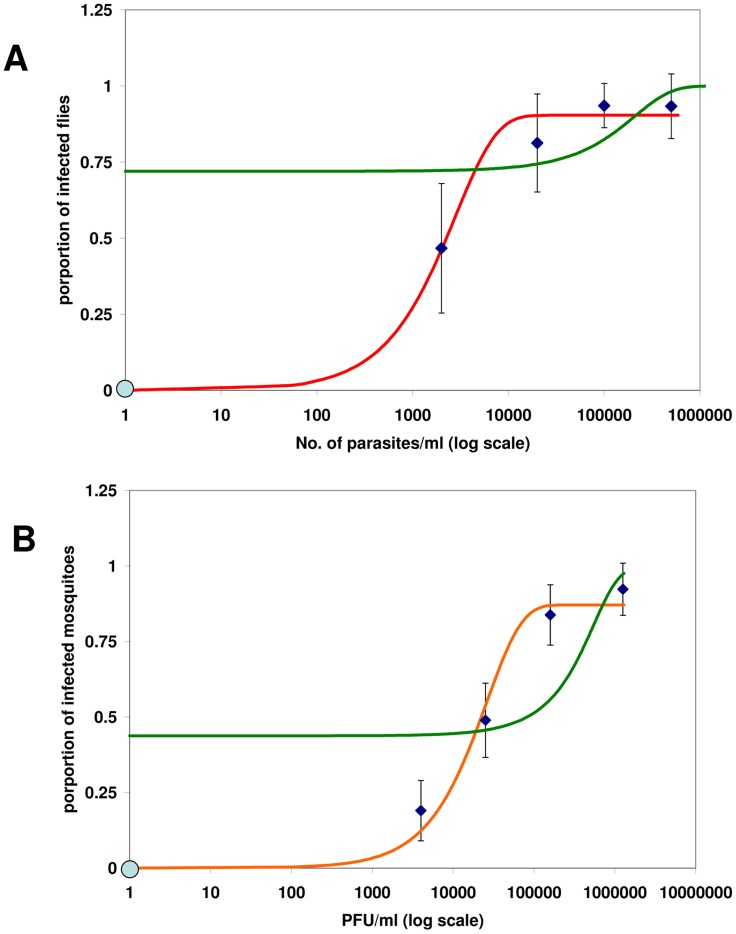
The infectiousness of blood with different parasite concentrations (parasitemias). We fitted the model (equation 4) and logistic function (y = 1/(1+exp[-λ_1_−λ_2_x])) to two different VBD set of results by maximum likelihood estimation (Nelder-Mead method) of the model parameters λ_1_ and λ_2_. The error bars in the proportion of infected vectors (y axis) were calculated as 95% confidence intervals of the respective binomial distribution. The origin in both panels (marked in blue) was taken as a data point, since we assume that a vector cannot be infected by uninfected blood. In red – our model fit, in green – logistic regression (A) The fitting results to data on VL [6]: our model: λ_1_ = 0.9037, λ_2_ = 3.58*10^−4^, logistic regression:λ_1_ = 0.9434, λ_2_ = 6.024*10^−6^ (B) The fitting results to data on Chikungunya [25]: our model: λ_1_ = 0.8712, λ_2_ = 3.82*10^−5^, logistic regression: λ_1_ = 0.2505, λ_2_ = 3.0482*10^−6^. PFU = Plaque-Forming Unit. Note that our model fits all data points within their confidence intervals. However, the logistic function is unable to fit the data points either for low parasitemia values (since it is always different than zero, thus the origin never belongs to its image), or for high parasitemia values (since it always approaches 1 for x→∞, although certain proportion of vectors can never be infected [17], [18], [19]).

Our infectiousness model (equation (4)) depends on two parameters; λ_1_, the infectious probability of a host with one parasite per ml, and λ_2_, the probability that a vector will be infected irrespective of the number of parasites ingested during blood feeding. First, the model parameters (λ_1_ and λ_2_, equation (4)) were estimated by performing nonlinear regression analyses of sand fly infection data obtained by artificially feeding *P. orientalis* sand flies with different concentrations of *L. donovani* parasites and determining their infection rates (Figure 1A) [6]. In order to demonstrate the generality of the model, we also estimated its parameters for previously published data on the infection rates of *Aedes aegypti* mosquitoes, the vectors of Chikungunya virus, after feeding on bovine blood with different virus concentrations [25] (Figure 1B).

The most common way to fit frequency data (e.g., infection rate, Figure 1) to a set of explanatory variables is through logistic regression [2]. The logistic function (*y* = 1/[1+exp(-*x*)]) links between a linear combination of the explanatory variables (*x*) and the frequencies that can only take values of positive fractions or zero (*y*). Yet, the logistic function and the linear combination of the model predictors may not always reflect reality correctly. A mechanistic model that relies on first principles and combines current knowledge of the respective systems may often be advantageous. In Figure 1 we also present the fitted results of the data sets of VL and Chikungunya to a logistic model (Figure 1, green lines). Clearly, the mechanistic model (equations (4)) has a better fit to the data sets of both diseases.

### Cohort Study

**Figure 2:**
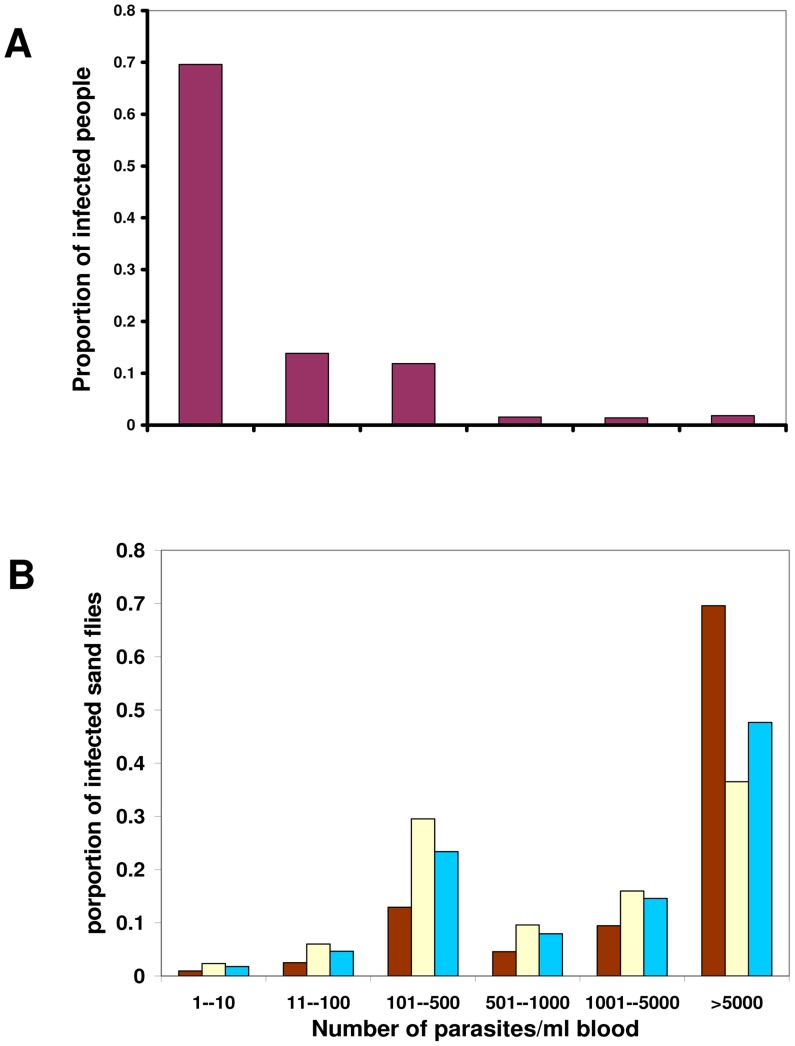
The contribution of asymptomatic carriers with different parasitemias to the infected sand fly population. (A) Division of the infected human population into parasitemia categories. Bars represent the proportion of the different parasitemias among the total infected human population (*N* = 658). Due to a large sample size (*N* = 658), the errors were of the order of 1% and hence negligible. (B) The calculated proportions (according to equation 5) of infected sand flies (from all infected sand flies) that were infected by feeding on individuals belonging to different parasitemia categories. Grouped bars represent the proportions of flies infected by biting people that belong to a particular parasitemia category (X axis). Different colored bars represent the proportion of infected sand flies (from all infected sand flies) for three different values of the model parameters, λ_1_ and λ_2_: mean, and the two edges of their 95% confidence intervals (the confidence intervals were calculated by parametric bootstrapping on Figure 1*A* data). Note that for high estimations of λ_1_ and λ_2_ (and hence *q*(*n*)), the relative contribution of people with low parasitemias to the population of infected sand flies would be larger compared to the case of low λ_1_ and λ_2_ (i.e., low *q*(*n*)).

From a cohort of 4,695 volunteers, 86% were kDNA-PCR negative (i.e. without parasites, in their blood, *n* = 4,037) while 14% (*n* = 658) were kDNA PCR positive indicating the presence of *Leishmania* parasites in their blood, rendering them potentially infectious to biting sand flies. The distribution of parasite concentrations in the infected population (14%, *n* = 658) is presented in Figure 2A. Figure 2A indicates that most infected individuals had very low parasitemias (∼70% had between 1–10 parasites per ml, *n* = 458), while very few had high parasitemias (3.2% had above 1,000 parasites per ml, *n* = 21).

### The Contribution of Hosts with Different Parasitemia Categories to the Infected Sand Fly Population

The distribution of the host parasitemias obtained from the cohort study (Figure 2A), and the estimation of λ_1_ and λ_2_ (Figure 1A), facilitated the calculation of the HIP (Figure 2B) via equation (5). Figure 2B indicates that persons with 1,000 parasites per ml or higher comprised only 3.2% of the infected human population, yet according to the model, they were responsible for the infection of between 53% and 79% (mean of 62%) of the infected *P. orientalis* sand flies (Figure 2B).

### Extension of the Model to Multihost Vector Borne Diseases

The model can easily be extended to facilitate the calculation of the contribution of a specific host species to the infected vector population in cases where the VBD is hosted by several species. To this end, we now formulate a more general form of equation (5) (which calculates the contribution of hosts with certain parasitemia range to the infected vector population, see methods), that can be used in cases of multihost VBD. Let *N* and *N_j_* be the number of host species involved in transmission and the number of individuals of species *j* (1≤*j*≤*N*) in a sample/cohort, respectively. The proportion of vectors that would be infected by feeding on species *j* from all infected vectors, *I_j_*:, is given by:
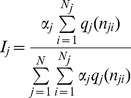
(6)


In equation (6), the numerator sums the contribution of all individuals of species *j* to the infection of the vector population, and the denominator sums that contribution of all individuals in the community. Note that here, unlike equation (4), *q_j_*, the probability that host species *j* would infect the vector as a function of its parasitemia, is species-specific, i.e., each host species is characterized by different λ_1*j*_ and λ_2*j*_, representing the λ_1_ and λ_2_ of species *j* (equation (4)). Thus, *q_j_*(*n_ij_*) is the probability that an individual *i* (1≤*i*≤*N_j_*) of host species *j* with parasitemia *n_ji_* would infect a naïve vector. Equation (6) is in fact a weighted average version of equation (5); the weights, α*_j_*, represent the vectors' preference towards species *j*. Numerous studies indicate that vectors may demonstrate preferences toward certain host species and even to specific individuals within the same species [1], [26], [27], [28]. The preference weights can be determined by attraction experiments, where the number of bites, or the number of vectors attracted to different host species are counted [29], [30], [31]. For example, if the numbers of vectors attracted to two host species (denoted by *r* and *s*) are *k*_r_ and *k*_s_, then:
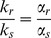
(7)Where 1≤*r*≤*N* &1≤*s*≤*N*. By using different combinations of equation (7) for all 1≤*r*,*s*≤*N*, the various preference weights α*_j_* (equation (6),(7)) can be calculated. Once these weights, λ_1*j*_, λ_2*j*_, and the parasitemia of each individual of species *j* in the sample, *n_ij_*, (1≤*i*≤*N_j_*) are measured, *I_j_* can be straightforward calculated via equation (6).

It should be stressed that the use of equation (6) is by no means restricted to multihost diseases. It can be used in any case where a host population/community can be divided into groups which vary in their attractiveness to vectors. In general, *N*, is the total number of host groups displaying distinct levels of attractiveness to vectors, *N_i_*, is the number of individuals in each group (group *i*), and α_i_ is the vector preference toward group *i* that can be determined experimentally via equation (7).

## Discussion

The model developed in this study facilitated the quantification of the contribution of hosts with different parasitemias to the infected vector population in VBD that are transmitted through blood sucking insects. In the case of *Leishmania donovani* carriers in northern Ethiopia, our model results indicate that the proportions of infected sand flies that became infected by feeding on persons belonging to different parasitemia categories (i.e., the HIP, Figure 2B) were substantially different from the proportions of these categories within the host population (Figure 2A). This difference can only be quantified by using the model (equations (4) and (5)). Our main finding was that only few infected people (about 3%) were responsible for the majority (about 60%) of the transmissions of VL in the region.

Heterogeneity in the contribution of different host groups to the transmission of infectious diseases has been phrased as the “20/80” rule, that is “20% of the hosts contribute at least 80% of the net transmission potential” [1]. However, the “20/80” rule refers to heterogeneity in contact rates between the disease infectious agents (i.e., hosts, vectors), and not to a difference in the infectiousness of different host groups for the vectors, as the current study does [1], [32]. This is an important difference, since the basic reproductive number, *R*_0_ (an important measure of disease risk) in this study, is not necessarily higher compared to a population with homogenous host infectiousness [1], [32].

As a property of the hosts, the HIP is unaffected by the vectors' biology. For example, temporal or spatial changes in vector abundance such as seasonal fluctuations or spatial heterogeneity in vector density, time delay between biting and infection that cause some vectors to die before infection is acquired [6], [20], and low temperature that reduces the success of the infection process within the vector body [20], may affect the total number of infected vectors, but not the host category they acquired their infection from (i.e., the HIP). The HIP will preserve its form (Figure 2B) even when the parasitemias of individual hosts change with time, as long as the parasitemia categories (Figure 2A) remain at dynamic equilibrium (i.e., the number of individuals that enter each category per unit time equals the number of individuals who leave it). When the parasitemia categories are not at dynamic equilibrium, the parasitemia distributions within the population (Figure 2A) and the resulted HIP (Figure 2B) will change with time, i.e., the contribution of each host parasitemia category to the infected sand fly population will be time dependent. The HIP is therefore a property which preservers under wide range of natural circumstances and spatio-temporal heterogeneities, a fact that makes it highly useful for studying the dynamics and control of many VBDs.

The presented model outlines a relatively straightforward and fast procedure for calculating the HIP (Figure 2B) by facilitating the prediction of the host infectiousness as a function of its parasitemia from the limited data often obtained by xenodiagnostic or artificial feeding experiments (Figure 1), and combining this prediction with the data on the distribution of host parasitemias within the population (Figure 2A). Furthermore, the model can easily be extended to quantify the contribution of different host groups with different attractiveness to the vectors (e.g., other host species) to the transmission of many multihost VBDs (equation (6)). According to previous surveys, these VBD types comprise the majority (>60%) of VBDs affecting humans (e.g., West Nile Fever, other types of Leishmaniasis, [33], [34], etc). When the vectors show preference toward a specific host group, the basic reproductive rate of the disease, *R*_0_, may increase, and consequently, so is the risk of disease outbreak and morbidity [35].

Our results imply that in the case of VL in northern Ethiopia, intervention focusing on a small fraction of the total population with high parasitemias (0.45% = 3.2% of the 14% infected humans), may substantially reduce the number of infected sand flies, and consequently the incidence of VL. Implementation of such targeted interventions is becoming more feasible thanks to the development of rapid mass screening techniques, such as those already recommended by the World Health Organization for prompt and accurate parasitological confirmation of malaria [36], [37], [38]. We stress that the mass screening demanded by our model can be both rapid and inexpensive, since it need not be accurately quantitative. It only needs to be sensitive enough to differentiate the hosts with high parasitemias from those with very low ones.

It should be stressed that the effectiveness of intervention strategies targeting the 3.2% of the hosts with highest parasitemias depends on the absolute size of the infected sand fly population; when the infected vector population decreases, so does the EIR (the Entomological Inoculation Rate, denoting the number of infectious bites an individual host receives per unit time), a well established index for disease persistence and outbreak [32], [39]. The basic reproductive rate, *R*_0_, of vector-borne diseases is proportional to the EIR, thus both indexes have similar properties; when the EIR is high enough, *R*_0_>1 (i.e., above the threshold value of *R*_0_ = 1) and a disease outbreak will occur. The higher is the EIR (or *R*_0_), the higher is the number of predicted infected hosts [32], [39]. The total number of infected sand flies depends on the HIP as well as the fly population dynamics (i.e., birth, death, and migration rates) that may vary in space and time (seasonality). If the birth and death rates are high, many infected vectors will die and be substituted with new-born susceptible ones. In such a case, although the HIP will be preserved (Figure 2B), the number of infected vectors and their proportion within the total vector population will be lower compared with a case where the population turnover is slow. The EIR also depends on the probability that a bite of an infected vector is infectious to the host. In this study, we discriminated between infected vectors (vectors with viable parasites in their gut) and uninfected ones. However, infected vectors may have different infectiousness levels [15], [40]. Thus, although a reduction in the 3.2% most infected hosts will decrease the total number of infected vectors, and consequently, reduce the *R*_0_, EIR, and morbidity, quantifying the success of such a control strategy necessitates the development of a dynamic model that involves data on the vector population dynamics and the distribution of the their levels of infectiousness. Such a model is beyond the scope of the current study.

Our study stresses the importance of the HIP due to its robustness to various natural circumstances. In turn, calculating the HIP helps to pinpoint the relevant host groups responsible for the transmission of different VBDs. Further development of dynamic models accounting for host parasitemia profiles (Figure 2A) that vary with time and vector population dynamics can broaden our understanding of the role of host groups with different parasitemias in the transmission dynamic of VL, as well as other VBDs.
